# “It Is Me Who Endures but My Family That Suffers”: Social Isolation as a Consequence of the Household Cost Burden of Buruli Ulcer Free of Charge Hospital Treatment

**DOI:** 10.1371/journal.pntd.0000321

**Published:** 2008-10-15

**Authors:** Koen Peeters Grietens, Alphonse Um Boock, Hans Peeters, Susanna Hausmann-Muela, Elizabeth Toomer, Joan Muela Ribera

**Affiliations:** 1 Partners for Applied Social Sciences|PASS International, Leuven, Belgium; 2 Institute of Tropical Medicine, Antwerp, Belgium; 3 Alphonse Um Boock, Aide aux Lépreux Emmaus-Suisse, Berne, Switzerland; 4 Leuven University, Leuven, Belgium; Centers for Disease Control and Prevention, United States of America

## Abstract

Despite free of charge biomedical treatment, the cost burden of Buruli ulcer disease (Bu) hospitalisation in Central Cameroon accounts for 25% of households' yearly earnings, surpassing the threshold of 10%, which is generally considered catastrophic for the household economy, and calling into question the sustainability of current Bu programmes. The high non-medical costs and productivity loss for Bu patients and their households make household involvement in the healing process unsustainable. 63% of households cease providing social and financial support for patients as a coping strategy, resulting in the patient's isolation at the hospital. Social isolation itself was cited by in-patients as the principal cause for abandonment of biomedical treatment. These findings demonstrate that further research and investment in Bu are urgently needed to evaluate new intervention strategies that are socially acceptable and appropriate in the local context.

## Author Summary

The cost burden of free of charge Buruli ulcer disease (Bu) hospital treatment is not sustainable for a majority of patients and their families in Central Cameroon. The long term nature of Bu taxes the patients' and their families' resources often to a breaking point, consequently often leading to the abandonment of patients by the family. In the study area, 62% of families ceased providing social and financial support to the patient, which resulted in the patient's isolation at the hospital. Significantly, social isolation was cited by in-patients as the principal cause for abandonment of biomedical treatment. Paradoxically, this phenomenon was observed in settings where hospital in-patient treatment, room and board were provided free of charge for the patient and caretaker. These findings show that despite the significant reduction in costs for medical care, in its current form, hospital treatment for Buruli ulcer often remains financially and socially unsustainable for patients and their households, leading to the abandonment of biomedical treatment or altogether avoiding it. Further investment and research are urgently needed to evaluate new intervention strategies that are both socially and financially acceptable and appropriate in local settings.

## Introduction

Buruli ulcer disease (henceforth, Bu) is the third most common mycobacterial disease in humans, after tuberculosis and leprosy [1]; nonetheless, despite the dramatic increase in incidence rates in West Africa during the last decade, it remains largely neglected [2]. Its causative agent, *Mycobacterium ulcerans*, is an environmental mycobacterium endemic to restricted foci throughout the tropics and directly related to stagnant or slow-flowing water [3]. Inoculation of *Mycobacterium ulcerans* into the subcutaneous tissues likely occurs through penetrating skin trauma, though the mode of transmission is not entirely clear. The agent produces a potent toxin known as mycolactone which destroys cells in the subcutis leading to the development of large skin ulcers [4]. Major advances have been made in the management of the disease with the introduction of rational antibiotic therapy [4], however, successful clinical outcomes require that patients initiate treatment promptly and adhere to treatment regimens [5],[6],[7]. Furthermore, because Bu often leads to irreversible physical disabilities, the disease takes a significant toll on affected patients and their households [7].

In Cameroon, Bu was first documented in 1969 in 47 cases, all originating from the well-circumscribed rural area of Ayos and Akonolinga in Central Cameroon. The endemic region was later identified as an area stretching along the Nyong River and some of its tributaries for approximately 100 km in length and 10–30 km in width with a population of 98,500 people [3]. A study by Noeske and others [3] conducted in 2001 identified an overall prevalence rate of 0.44% constituting active and inactive Bu in the surveyed area. The highest prevalence of active cases found in a particular settlement was 8%. Disease prevalence was higher in villages closer to the Nyong river. Bu occurs principally among impoverished rural people with limited geographic and economic access to health facilities. Recently, a survey carried out by Um Boock in 2004 [8] detected new foci of Bu outside of the previously established endemic region, particularly in other areas of the Central Province and in the provinces of the East and Southwest. At the national level 930 cases were detected [8].

Biomedical treatment Bu in the endemic region is provided at the Ayos and Akonolinga Hospitals which have specialised Bu programmes sponsored by *Aide aux Lépreux Emmaus Suisse* and *Médicins Sans Frontières* respectively. Both offer free of charge medical treatment and supplementary aid. These services include free of charge medication and in-patient treatment; free meals served once or twice a day (depending on the institution); complementary accommodations for in-patients and their caretakers for the duration of their stays; extra schooling (at Ayos Hospital); and, the free provision of basic materials for everyday needs such as soap and bandages, sheets, etc. However, the provision of these materials is often irregular as stock-outs are common, directly affecting patients' hospitalisation costs.

The main objective of this article is to evaluate the economic and social impact of hospital treatment for Bu disease on the patient and the household in a setting where medical costs for hospital treatment and supplementary aid for everyday needs are subsidised by the local health care system and/or through foreign aid.

## Methods

### Field work and participant observation

The present study was conducted in the above-mentioned endemic region of Ayos and Akonolinga. It was an a focused ethnographic study using both qualitative and quantitative research methods. Field work was conducted in both community and clinical settings for a period of four months, three of which were spent at the Ayos and Akonolinga hospitals and one in the selected endemic communities of Eyess, Edou, Ebanda and Ngoulemakong, all belonging to the catchment areas of the respective hospitals. Considering the focalised character of Bu infection rates, [7] restricted local and geographical units were selected for analysis.

During field work the following techniques were used: (i) *Participant observation*. Participant observation, or the observation of people's behaviour in its natural setting, is a fundamental and often neglected part of qualitative research. This technique consists of participating in everyday activities, working in the native language (in this case the official language and *lingua franca*, French) and observing events in their everyday context. Gaining patients' and household members' confidence through participant observation methods increases the validity of ethnographic data. In this study, participant observation was particularly important in establishing costs since patients and household members are unlikely to accurately recall all costs associated with this long-term illness. Participant observation also enabled us to contrast reported adherence to treatment, expenditures, frequency of visits to hospitalised Bu patients, and other behaviours with actual observed patterns. (ii) *In-depth interviews*. During field work, in-depth interviews were carried out with Bu patients and their households, extended family members, local traditional healers, community leaders and key informants (such as teachers, school directors, priests, etc). One or more in depth interviews were carried out with Bu patients and their households. All interviews were conducted by the authors, personally, and were not mediated by hospital, public health or other biomedical staff for fear that their presence would guide responses (e.g. downplay negative opinions of current health provisions). (iii) *Focus group discussions*. Focus group discussions were held with medical staff at the Ayos and at the Akonolinga Hospitals and with extended family groupings in the various communities. Treatment, costs and health seeking behaviour (including perceived aetiology of the illness, traditional healing and delay) were among the primarily topics during these sessions.

Insights and established categories from an initial phase of qualitative research were used to assess preliminary data, and construct cost categories, which facilitated further systemisation and eliciting of costs from respondents, and were useful as secondary indicators to evaluate people's stated costs and economic coping strategies.

After this stage, data were more systematically gathered and standardised through (iv) a quantifiable *half-open structured questionnaire* realised with all patients at Ayos and Akonolinga Hospitals and carried out in the local communities. At the Ayos and Akonolinga Hospitals, 79 clinically confirmed hospitalised Bu patients were included in the sample, representing all patients in treatment during the four month period of the study from November 2005 to February 2006. The costs and cost burden presented in this article apply exclusively to the hospitalised patients. However, to gain a better understanding of Bu patients' and households' health seeking behaviour, the social and economic burden of the disease, and the relationship between local communities and the hospital setting, 73 patients and their households were further included for qualitative analysis at the community level, representing all active and inactive Bu cases living in the selected communities at the time of study. The costs accrued by the latter are outside the scope of this article.

#### Data Analysis

All interviews and a record of field observations and important informal conversations were coded in NVivo Qualitative Analysis Software (QSR International Pty Ltd. Cardigan UK). NVivo enables the management and coding of large sets of qualitative data, and facilitates linking them to quantitative data management and analysis software. Further quantification and analysis was carried out in SAS (SAS Institute Inc., SAS Campus Drive, Cary, North Carolina 27513, USA).

### Concept Definitions

#### Social Isolation

Costs for socially isolated and socially not isolated patients were included in the study after preliminary qualitative research. Social isolation was understood as a sudden or progressive loss of social relations, family or friends that form the patient's social network. For the category ‘Children & Adolescents’ social isolation was defined as being hospitalised without caretaker, and for ‘Adults & Elderly’ as having no caretaker *and* receiving less than an average of 1 visit every 15 days. Both definitions were operational: The absence of a caretaker is a defining characteristic of abandonment and social isolation for children and adolescents in the study setting as were the supplementary number of visits used to define social isolation for adults and elderly. Qualitative data and secondary indicators (such as patients stating they are “abandoned”; being hospitalised in “solitude”; having no social networks for supplementary costs and food provisions) affirmed the validity of the definitions in the local context.

### Cost Categories

**Figure 1:**
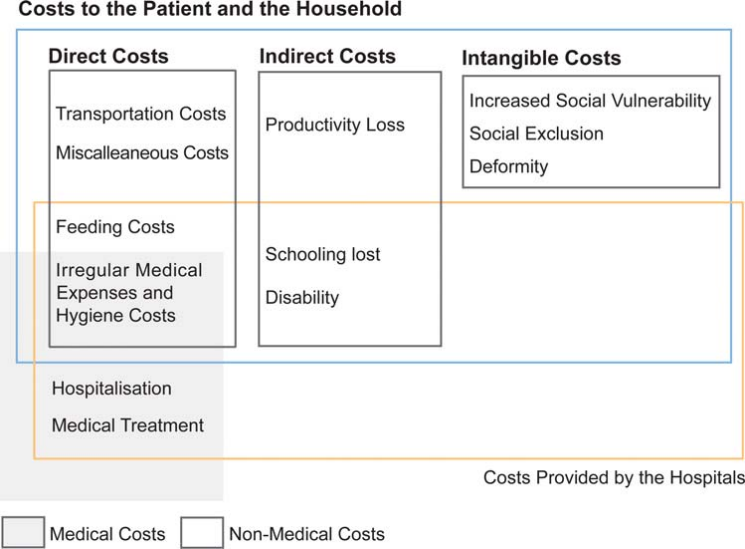
Costs to the Patient and the Household.

For the definition and categorisation of costs, the conceptual framework proposed by Russell [9] was employed to ensure clarity and precision and to allow for further theory building and comparability of the economic burden of the illness across settings (Figure 1; Table 1).

**Table 1:** Systematic Overview of Cost Definitions.

Cost Categories	Definition
Cost burden	Percentage of household earnings consumed by total costs of treatment
Total Costs	Direct costs plus indirect costs of treatment
Direct Costs	Expenditures incurred by the patient or household members in the course of treatment
Direct Costs of Patient's Care	Expenditures by the patient or household members that are directly related to the patient's treatment
Direct Costs of Household Members' Involvement	Expenditures by the patient or household members that are directly related to the involvement of household members in the patient's treatment
Indirect Costs	Value of productivity or earnings lost by the patient or household members during the course of treatment
Indirect Costs of Patient's Care	Value of productivity or earnings lost by the patient or household members due to the patient's treatment:
	(1) Lost productivity (adults) - value of patient's lost productivity during treatment
	(2) Lost schooling (children) - value of patient's lost schooling during treatment
Indirect Costs of Household Involvement	Value of household members' lost productivity during treatment

#### Medical and non-medical costs

In the present setting, the medical costs of Buruli ulcer disease in-patient treatment at the Ayos and Akonolinga Hospitals are largely covered; therefore the costs patients and households face are almost exclusively *non-medical* (minor exceptions include extra medication bought by patients for pain relief, extra bandages, etc. due to the mentioned regular stock outs at the hospitals or the perceived necessity of patients to purchase extra medication).

#### Direct Costs

Patient and households' ‘Direct Costs’ were understood as expenditures incurred by the patient or household members in the course of treatment. For analytic purposes, systematically included Direct Costs were divided in the following categories: (i) *Irregular Medical Expenses and Hygiene Costs*. These costs are related to Bu patients' personal hygiene when taking care of the ulcer (e.g. bleach and soap to wash bandages and clothing dirtied due to the ulcer); irregular expenses for extra medication (i.e. for pain relief) and unofficial fees during treatment. (ii) *Feeding Costs*. Feeding Costs include extra meals taken at local food stands by patients or caretakers; (iii) *Transportation Costs*. Transportation Costs refer to the costs of transport for the patient, caretaker(s) and other household members when travelling to and from the hospital to visit the hospitalised patient; (iv) *Miscellaneous Costs*. These include a variety of non-systematic costs such as extra rent in the location of the hospital for caretakers, extra phone calls, debts to community workgroups due to illness, gifts to hospitalised patients, etc. For all the preceding costs calculations, changes in the use of stream of resources were measured.

#### Indirect Costs

‘Indirect costs’ refer to the value of lost productivity or earnings lost by the patient or household members in the course of treatment. Productivity lost was based on the calculation of the individual's (patient and/or caretaker) earnings per calendar year and the percentage of these earnings that was lost due to the morbidity time caused by the illness episode or caretaking. For children and youth, additional schooling lost due to Buruli ulcer disease was evaluated.

#### Total Costs

Total Costs represent the sum of the Direct Costs and the Indirect Costs of the illness.

#### Cost Burden

The ‘Cost Burden’ refers to the percentage of the total household earnings that was consumed by Bu treatment costs. For this study, the cost burden of the illness was calculated based on (i) each individual patient's ‘total illness costs’ during hospital treatment as a percentage of (ii) the yearly earnings of his or her household (for more details see also *Prices and Currencies* and *Illness Costs* below).

#### Cost of Patient's Care and Cost of Household Members' Involvement

In each of the preceding cost categories, cost of patient's care was distinguished from cost of household members' involvement. Cost of patient's care was defined as expenditures or lost productivity by the patient or household members that are directly related to the patient's treatment. Cost of household involvement was defined as expenditures or lost productivity by the household that are related to involvement of household members in the patient's treatment.

### Cost Calculations

#### Prices and currencies

Due to (i) the limited time period over which costs were calculated (median in-patient hospitalisation time was 157 days; household earnings were calculated on a yearly basis); (ii) the difficulties of having reliable economic indicators adapted to the local setting; and (iii), the absence of extreme inflation (in 2005, inflation rates were 4.7%), *current* prices were employed in cost calculations. Prices refer to those valid during the second part of the year 2005 and the first part of the year 2006 (conversion rates used are from 04/2006, otherwise €1 = 655,95 FCFA, €1 = $1.19).

#### Illness costs

Patients were followed up during the four month period at the hospital and interviewed regularly to assess all costs of the illness during hospital treatment. All resources the patients and their households used were quantified using established variables and categories from initial qualitative analysis, which included (i) a table presenting the different kinds of commodities used by the patients and households; (ii) the respective prices of those resources (and secondary verification of purchased product prices) (such as soap, bandages, etc.); and, (iii) the quantity of resources used.

For patients that were discharged before the end of the study, all costs were calculated from the beginning of their hospital treatment (including initial seeking of hospital treatment) until the day of discharge; therefore, all costs were retrospective. For patients still in treatment after the four month study period, total costs were extrapolated to the median in-patient treatment time for patients at the Ayos and Akonolinga hospitals since the Bu programmes started in 2002, which equalled 157 days.

Median values are preferable for cost burden research (as is recommended by Russell, [9]) as they avoid outliers and, as such, give a more accurate idea of the economic burden then average values. For the desegregation of total costs, in order to evaluate the respective weight of its subcategories (such as Direct & Indirect Costs), the mean was preferred for its statistical characteristics.

### The Household as Unit of Analysis

In the present context, the household was operationally defined as a group of people who live together in a common residence, forming a unit of economic cooperation, and who are responsible for the socialisation of the children born of its members. The household was the preferred unit of analysis for assessing the economic costs and consequences of illness for two reasons. Firstly, local kinship relations are traditionally based both on patrilineal filiation (kinship through the father's line) and patrilocal residence patterns (adoption of the father's place of residence in marriage). Communities are consequently divided into different settlements of patrilocally nucleated extended family clusters (or *patrikin* groups), consisting of various related households that nevertheless function *independently*. Despite the proximity of *patrikin*, the household is the basic economic unit when coping with the illness costs of its members. Secondly, because decisions about treatment are based on negotiations within the household (though not necessarily from an equal bargaining position), since the costs of illness reach beyond the sick to involve other household members who care for and accompany them to treatment and who ultimately are affected by the costs of illness which fall on the household budget and diminish the resources available for other household members [9].

### Household Earnings

Household subsistence strategies at the local level consist of a combination and alternation of revenues. Of primary importance is slash and burn agriculture, characterised by the simultaneous exploitation of several plots with a variety of intercropped products (such as plantains, macabos, maniocs and peanuts) that are harvested at different periods and can be more intensively exploited according to the necessities of general household spending. Earnings from slash and burn agriculture are often supplemented by earnings from cacao and/or café plantations (a heritage of colonial times), which require different agricultural techniques and can only be harvested at specific intervals each year. Furthermore, the proximity of the Nyong and its tributaries provides communities with fish while the tropical rainforest offers possibilities for hunting. Both resources are used for consumption and sale. Occasional formal and salaried employment and migration to urban centres or abroad represent additional complementary subsistence strategies, although the latter signifies the separation of the household.

Earnings were compiled through in-depth interviews with the household providers and was calculated based on the combined earnings of all household members for the period of one calendar year. For most cases, households were entirely dependent on subsistence farming. For slash and burn farming, activities were systemised in an agricultural calendar specifying products' harvest times or intervals, product prices and the subsequent estimation of earnings. Earnings from fishing were calculated according to an activity calendar, incorporating the amount of fish, fish prices and number of days worked. Hunting is a more irregular activity and meat is consumed more often then sold. Nevertheless, extra earnings of sold bush meat were also incorporated into calculations. In cases where one of the household members had formal employment, his/her yearly earnings were calculated according to the official salary. Usually this person further participates in the household's slash and burn agriculture, the revenues of which contribute to the general household earnings. The yearly revenues for informal urban and semi-urban employment (i.e. undocumented, unofficial and irregular employment such as construction work, etc.) were also included in calculations for the patient and/or other household members.

### Methodological Limitations

#### Costs not included

The following costs have not been included: (1) *Time costs of visitors*. Visitors' time costs were not included because they generally use days and hours with minimal work obligations to visit hospitalised patients and therefore lose less productive time making the value of this time loss too abstract to include in productivity loss calculations. (2) *Extra irregular expenses*. Patients, caretakers and visitors tend to make unquantifiable purchases on their way to and from the hospitals, such as food and drink for the journey that can not practically be incorporated into cost assessments. (3) *Locally produced products*. Food products harvested by farmers in their local communities and given to the patient on visits have not been included in cost calculations as these products do not represent a direct monetary expense for the family. Moreover, due to the irregularity of food provision for the patient and the frequent use of such products for purely consumptive rather than economic purposes, it is difficult to express this dynamic economically.

#### Incalculable or Intangible Costs

Certain consequences of Bu cannot be expressed or directly converted into monetary values. These include some of the illness's long term consequences such as abandonment of schooling; disability and deformity; social exclusion, and psychosocial factors, the effects of which can not tangibly be measured, but that are, nonetheless, detrimental to the patient and the household. An example of such intangible costs during treatment is the increased social vulnerability due to reduced participation in local social security networks. The patient's and the household members' absence from the community results in enhanced vulnerability in times of crisis due to their inability to maintain participation in community work groups and savings clubs that require regular contributions. In this respect, absence from the community leads to increasing vulnerability and potential impoverishment since participation in those structures can assist in recovery during such periods.

#### Limitations

The long term nature of the illness inevitably leads to the following methodological limitations in the calculation of costs: (i) *Earnings*. The loss of earnings was calculated on the basis of the earnings of the patient when he/she stopped working due to Bu hospital treatment. However, the long term nature of the illness makes it impossible to measure indirect loss in terms of previously missed opportunities in schooling or work. In other words, it is impossible to assess the entire extent to which an individual's or household's income has been altered by Bu and its influence on patients' present occupations. This is especially relevant as a high percentage of patients had active Bu for over 5 years and 10% of patients for over 10 and up to 32 years before receiving treatment in a specialised Bu-programme. (ii) *Recall period*. Unlike illnesses with brief and intense episodes, such as malaria, the long-term nature of Buruli ulcer disease diminishes the accuracy of patients' recall of all costs incurred and the exact value of those included. Therefore, costs are likely an under-estimate of the real costs accrued when coping with Bu. (iii) *Internal variance*. The cost burden of Bu significantly varies according to a number of factors, including the stage of illness when patients arrive at the hospital, but also in relation to social and economic status, medical complications, the distance between the community and the hospital, the availability of funds in relation to the needs of other household members, etc. However, it is beyond the scope of this article to go into detail about costs related to each category. Rather, the article emphasizes the relationship between costs, cost burden, adherence and social consequences at the local level.

### Informed consent

The study was approved by the ethical committee of the Ministry of Health, Cameroon (No. 0123/ARRO/MSP/DPSPL). For the field work, all interviewers were requested to follow the Code of Ethics of the American Anthropological Association (AAA) [10]. As proposed by the AAA, all interviewees were informed before the start of the interview about project goals, the topic and type of questions, their right to reject being interviewed, to interrupt the conversation at any time, and to withdraw any given information during or after the interview, and the intended use of results for scientific publications and reports to health authorities. Anonymity was guaranteed and confidentiality of interviewees was assured by assigning a number to each informant. The interviewers sought oral consent from all interviewees. Oral consent was preferred, since the interviewees were not put at any risk of being harmed in their safety or psychological well-being and the act of signing one's name when providing economic data was considered a potential reason for mistrust (see also AAA which states that “It is often not appropriate to obtain consent through a signed form-for example (…) where the act of signing one's name converts a friendly discussion into a hostile circumstance” [11].

## Results

### In-patient Treatment Time

During the study period, 34% of all patients registered at the Ayos and Akonolinga Hospitals since 2003 were classified as “healed” with a median hospitalisation time of 157 days (range 47–645 days). 39% of hospital patients were female and 61% male. 56% were children and adolescents (<20) while 44% were adults or elderly.

### Patient and Household Earnings

Monthly household earnings of Bu hospital patients previous to their illness were calculated for all patients and households. The median value was €40,7 (26.400 FCFA). This was slightly higher than the median earnings for subsistence farming in the studied endemic communities, which was estimated during fieldwork at €33,8 (22.000 FCFA). These median values correspond with those of the World Bank [12] and UNDP [13], which state that 51% of Cameroon's population lives below the $2/day mark while 17% earn less then $1/day.

### Costs per Category & Cost Burden

#### Total Costs

Median total costs of hospital treatment were €126,7 (82.372 FCFA) (table 2).

**Table 2:** Median Costs per Category*.

Cost Categories	Median Values (in €**)
Total Costs	126,7
Direct Costs	59,3
Direct Costs of Patient's Care	25,2
Direct Costs of Household Members' Involvement	33,7
Indirect Costs	64,4
Indirect Costs of Adult Patient's Care	158,4
Indirect Costs of Household Members' Involvement	106,9

*: Median costs are reported to avoid the influence of outlying values. Thus subcategories will *not* sum to unity.

**: Exchange rate of 04/2007. €1 = 655,95 FCFA , €1 = $1.19.

#### Direct Costs

Median direct costs totalled €59,3 (38.563 FCFA). The distribution of the direct costs shows that 19% is spent on Medical & Hygiene costs (including soap, bandages, extra medicine, etc.), 29% on Transport, 25% on Feeding Costs, and 28% on Miscellaneous Costs. The distribution of median costs shows that 43% (€25,2) stemmed from expenses directly related to the costs of the patient's care while 57% (€33,7) derived from household members' involvement during the illness period.

#### Indirect Costs

The median indirect costs of the patient's care (including both productive and dependent members of households), was €64,4 (43.809 FCFA). The median cost of productivity time lost during the patient's care for adult patients was €158,4 (102.949 FCFA). For children and youth, additional schooling lost was measured. Of all hospitalised Bu patients, 88% of children had lost schooling due to their illness; 20% of these had completely abandoned school while 68% had lost a median of 1 year of schooling (range 1–5 years; average; 1,71 years). *Indirect costs of household members' involvement:* The median productivity time lost for household members' involvement (when applicable) equalled €106,9 (69.489 FCFA).

#### Cost Burden

The median cost burden of Bu amounted to 25% of household's annual earnings. Direct costs represented 8% of patients' and households' yearly earnings while indirect costs accounted for 17%. Therefore, 31% of the cost burden total costs were direct while 69% were indirect.

### Coping and Cost Prevention Strategies

During in-patient treatment, the most common coping strategies included the use of savings (39%); making claims from social networks (75%); borrowing (53%); reducing consumption of non-essentials (100%) and essentials (69%); patient informal employment (36%); informal employment of the caretaker (43%); the supplying of provisions from family relations in nearby villages (56%); and, the social isolation of the patient (63%). The coping strategies employed were generally cost prevention strategies, similar to those used for other long-term and chronic illnesses, examples of which are found in studies of TB illness costs where households engage in either cost prevention strategies (do not seek treatment or abandon treatment) or asset strategies to mobilise substantial sums of money [9],[14],[15].

### Patients' Social Isolation

The median total costs for household members' involvement for a patient that was not isolated totalled €105,9 (68.848 FCFA) while for an isolated patient the costs decreased dramatically to only €12,4 (8.051,87 FCFA). These numbers signify that for patients who were not isolated the costs for household involvement during the healing process was 8,6 times higher than for isolated patients. Subsequently 63% of households did avoid these costly direct expenses, leading to the social isolation of the in-patients. In the words of isolated patients:

*- None of my brothers have come to visit me here, not even my children. I don't know if it's the costs of transport, whether that is too much for them or if they have neglected my person. They await me or my dead body. I have taken my heart away from the village. Now that I'm here I only think about the hospital. I don't know what's going on in my village. I no longer care. But when I'll return to the village I will still be me and I will live with them.*
*- My son no longer comes to visit me, I don't know if he doesn't even consider me his father any more, because if he did he should be here.*
*- I told my children not to come anymore, it's better for them to spend the money in the village. It's a ruin for them to visit me.*

## Discussion

Growing awareness of the connection between ill health and impoverishment has placed health at the centre of development agencies' poverty reduction targets and strategies and strengthened arguments for a substantial increase in health sector investment. Subsequent research, however, has shown the difficulty in alleviating the cost burden of illness for poor households due to the presence of *hidden* costs. Our research found that despite the availability of free of charge medical care, households of Bu patients in the study area faced an unbearable cost burden. The majority of households responded by withdrawing involvement in care, leaving patients socially isolated. This reality raises questions of how to increase accessibility to biomedical treatment and how to proceed with future programs.

The median cost burden of Bu hospital treatment was estimated at 25% for costs directly covered both by the household and by the patient. In most illness studies mean direct costs are estimated between 2,5% and 7,0% of household earnings. However, many chronic illnesses, such as TB in Malawi, account for cost burdens between 8–20% of annual earnings; and in sub-Saharan Africa and Thailand, AIDS related treatment absorbed 50% or more of annual income [9] (median values were not presented in the referenced articles).

With its 25% cost burden, Bu surpasses the cost burden threshold of 10%, which has shown to be catastrophic for the household economy [9],[16],[17] and likely leads to further impoverishment. Households, therefore, recur to a variety of coping strategies to manage these expenses and indirect losses. A common coping strategy against the accumulation of unmanageable costs is manifest in the breaking of ties with the individuals most taxing on the household economy; in this case the patients. As stated in the results, of the total direct cost, only 43% are expenses directly related to the patient and his or her treatment, while 57% are due to household involvement during the illness period. However, the 57% of direct expenses can -*and often is*- avoided by households. This circumvention of costs was evident for 63% of in-patient children and 71% of the adults who had no caretaker present. These numbers signify that for patients who are not isolated the direct costs for their households during the healing process is 8,6 times higher than for isolated patients.

Furthermore, qualitative research affirmed that the caretaker during hospital treatment is not a stable fixture; rather, a considerable percentage of caretakers leave during treatment, especially when they perceive the illness to no longer be ‘serious’. Subsequently, 63% of in-patients were socially isolated at the hospital setting. Furthermore, initial qualitative data showed that, according to hospital patients, social isolation is the principal cause for abandonment of in-patient treatment. In endemic communities, it was further stated that fear of social isolation is one of the major reasons for postponing or avoiding hospital treatment and why traditional healing is often preferred since it is usually locally provided or provided in the vicinity of the patient's community.

With respect to the cost burden equation, the weight of certain variables such as transportation costs, feeding costs and productivity loss of caretaker(s) are proportionate to the *distance* from the community to the treatment centre: (1) *Transportation costs*. While transportation costs are an additional cost for the patient, they represent a debilitating burden for caretakers since they continue to have social obligations to other household members (i.e. other children), signifying repeated visits to the hospitalised patient. Accordingly, transportation costs account for 29% of median direct costs. (2) *Feeding costs.* The costs of food for the patient at the hospital setting can be minimised through the provision of agricultural products from the household's slash and burn cultivation. However, this coping strategy is only feasible in cases when regular visits are also feasible, such as when treatment is carried out in the general vicinity of the patient's community. This avoids extra expenses for paid meals for patients and caretakers (feeding costs during hospitalisation represent 25% of median direct costs). (3) *Productivity loss*. Though the productivity loss of Bu patients could arguably be comparable in the hospital and community settings, that of the caregivers is greatly affected by the location of patient's treatment. When the patient receives treatment in the community or within the vicinity the caregiver can combine his/her daily economic activities with the patient's care without a serious impact on productive activities (median indirect costs for caretakers represent one of the most taxing costs).

The importance of the above-mentioned factors in relation to the economic and social impact of Buruli ulcer disease was also evident in patients' health seeking behaviour during field work. The fact that traditional healing minimised or largely avoided such costs was cited by respondents as a major reason why traditional treatment was often preferred to biomedical treatment. In this sense, decentralisation is expected to sidestep or drastically reduce the mentioned costs and to increase the sustainability of households' involvement during the patient's illness period, reducing patients' social isolation and create possibilities to increase adherence.

From a socio-economic perspective, it can, therefore, be concluded that a decentralised system of treatment with minimal hospital stays could limit household impoverishment as the long term nature of the illness makes it impossible for a household to indefinitely sustain its involvement with the hospitalised patient. A multidisciplinary study to evaluate a decentralised system of care with minimal hospital stays is, therefore, essential.

## Supporting Information

Alternative Language Abstract S1 Translation of the Abstract into Spanish by Joan Muela Ribera
(0.03 MB DOC)
