# Correction: “It Is Me Who Endures but My Family That Suffers”: Social Isolation as a Consequence of the Household Cost Burden of Buruli Ulcer Free of Charge Hospital Treatment

**DOI:** 10.1371/annotation/dbad2cfd-55a6-4f2e-b7eb-a37b0e989472

**Published:** 2008-10-22

**Authors:** Koen Peeters Grietens, Alphonse Um Boock, Hans Peeters, Susanna Hausmann-Muela, Elizabeth Toomer, Joan Muela Ribera

There was an error in affiliation number 3. The correct affiliation is: Aide aux Lépreux Emmaus-Suisse, Berne, Switzerland. 

