# Correction: “It Is Me Who Endures but My Family That Suffers”: Social Isolation as a Consequence of the Household Cost Burden of Buruli Ulcer Free of Charge Hospital Treatment

**DOI:** 10.1371/annotation/5a338934-9fe1-43be-9b8d-cbf3901ecbf5

**Published:** 2008-11-26

**Authors:** Koen Peeters Grietens, Alphonse Um Boock, Hans Peeters, Susanna Hausmann-Muela, Elizabeth Toomer, Joan Muela Ribera

The author affiliations were displayed incorrectly for the second and third authors. Alphonse Um Boock should be listed with affiliation number 3 (Aide aux Lépreux Emmaus-Suisse, Berne, Switzerland) and Hans Peeters with numbers 1 (Partners for Applied Social Sciences|PASS International, Leuven, Belgium) and 4 (Leuven University, Leuven, Belgium).

