# Supplementary material for: “It Is Me Who Endures but My Family That Suffers”: Social Isolation as a Consequence of the Household Cost Burden of Buruli Ulcer Free of Charge Hospital Treatment
Source: PLoS Negl Trop Dis. 2008 Oct 15;2(10):e321. doi: 10.1371/journal.pntd.0000321 (PMC2562517; doi:10.1371/journal.pntd.0000321)
Supplement: Alternative Language Abstract S1 — Translation of the Abstract into Spanish by Joan Muela Ribera (0.03 MB DOC) [file pntd.0000321.s001.doc]

**“Yo soy quien aguanta la enfermedad, pero mi familia es quien la sufre”: El aislamiento social como consecuencia de la carga económica de la úlcera de Buruli para las unidades domésticas**

La úlcera de Buruli tiene curación, siempre y cuando los pacientes inicien el tratamiento en su estadio inicial y lo cumplan de manera sistemática. De todos modos, es habitual que los pacientes tiendan a llegar al hospital en una fase avanzada de la enfermedad (Aujoulat *et al.* 2003; Stienstra, 2005). Como dolencia de larga duración, que a menudo provoca distintas formas de incapacidad física irreversible, la úlcera de Buruli supone un elevado peaje tanto para los pacientes como para sus unidades domésticas (Johnson, *et al.* 2005). Sin embargo, poco sabemos del impacto social de la úlcera de Buruli, así como de su carga económica para las unidades domésticas.

*Objetivos y métodos*

En este artículo estudiamos el impacto social y la carga que supone la úlcera de Buruli para la economía doméstica en Camerún. Estudiamos un total de 152 casos: (1) En los hospitales de Ayos y Akonolinga incluimos 79 casos clínicos confirmados de pacientes hospitalizados a causa de la úlcera de Buruli, esto es, el total de pacientes tratados durante los cuatro meses de trabajo de campo (de noviembre del 2005 a febrero del 2006). (2) En las comunidades de Eyess, Edou, Ebanda y Ngoulemakong incluimos a todas la personas – 73 - con úlceras de Buruli que en el período del estudio residían en dichas comunidades. Todos los casos, activos o inactivos, de úlcera de Buruli fueron registrados y entrevistados en profundidad. Asimismo realizamos entrevistas en profundidad con familiares de pacientes e informantes clave (como sanadores tradicionales, líderes comunitarios, maestros, sacerdotes, etc.). Para todos los pacientes calculamos los costes directos e indirectos del tratamiento hospitalario, tanto para ellos como para la unidad doméstica.

*Resultados*

A pesar del tratamiento hospitalario gratuito proveído en los hospitales de Akonolinga y Ayos, la carga económica de las hospitalizaciones es enorme, representando el 25.2% del ingreso anual de las unidades domésticas. Estos costes tan elevados proceden, sobre todo, de costes poco evidentes, particularmente de efectos no médicos y costes indirectos (aquí seguimos la definición de Russell, 2004). En el 62.0% de los casos hospitalarios los elevados costes no médicos y la pérdida de productividad para las unidades domésticas llevaron al aislamiento social de los pacientes, puesto que la implicación de los miembros de las unidades domésticas en el proceso terapéutico no era sostenible. Para aquellos pacientes que mantuvieron el contacto con sus familiares los costes directos para sus unidades domésticas durante el tiempo de la cura fue 8.5 veces más alto que para los pacientes aislados. El aislamiento social fue la principal razón de abandono del tratamiento biomédico por parte de los pacientes ingresados.

*Conclusiones*

Unos gastos del 25.2% de los ingresos anuales claramente sobrepasan los límites considerados catastróficos para la economía doméstica, generalmente estimados sobre el 10.0%. Estos resultados apoyan, desde una perspectiva socio-económica, la necesidad de potenciar el control de la úlcera de Buruli en las regiones donde es endémica. Demuestran además la necesidad de revisar las estrategias actuales, basadas en el tratamiento hospitalario y desarrollar nuevas intervenciones socialmente aceptables, adecuadas al contexto local. En nuestra área de estudio estas estrategias deberían incluir la descentralización de las curas especializadas y una máxima reducción de los ingresos hospitalarios.

# Referencias

Ajoulat, I., Johnson, C., Zinsou, C., Guédénon, A. and Portaels, F. (2003). Psychosocial aspects of health seeking behaviours of patients with Buruli ulcer in southern Benin. *Tropical Medicine and International Health 8* (8), pp. 750-759.

Johnson RC, Sopoh GE, Boko M, Zinsou C, Gbovi J, Makoutode M, Portaels F. Distribution of *Mycobacterium ulcerans* (Buruli ulcer) in the district of Lalo in Benin. *Trop Med Int Health*. 2005 Sep;10(9):863-71

Russell, S. (2004). The Economic Burden of Illness for Households in Developing Countries: A Review of Studies Focusing on Malaria, Tuberculosis, and Immunodeficieny virus/acquired immundeficiency symdrom. *Am. J. Trop. Med. Hyg., 71*, 147-155.

Stienstra, Y. (2005). *Mycobacterium ulcerans disease in West Africa*. PhD Thesis. Groningnen University, The Netherlands
